# Observational study on Swedish plaque psoriasis patients receiving narrowband-UVB treatment show decreased S100A8/A9 protein and gene expression levels in lesional psoriasis skin but no effect on S100A8/A9 protein levels in serum

**DOI:** 10.1371/journal.pone.0213344

**Published:** 2019-03-13

**Authors:** Albert Duvetorp, Jan Söderman, Malin Assarsson, Marita Skarstedt, Åke Svensson, Oliver Seifert

**Affiliations:** 1 Department of Dermatology and Venereology, Division of endocrinology, skin, reproductive health and ophthalmology, Skåne University Hospital, Malmö, Sweden; 2 Department of Clinical and Experimental Medicine, Faculty of Medicine and Health Sciences, Linköping University, Linköping, Sweden; 3 Laboratory Medicine, Division of Medical Diagnostics, Ryhov County Hospital, Jönköping, Sweden; 4 Department of Dermatology and Venereology, Division of Medical Health, Ryhov County Hospital, Jönköping, Sweden; Kinki Daigaku, JAPAN

## Abstract

S100A8 and S100A9 proteins are highly upregulated in patients with psoriasis and have been proposed as potential biomarkers for psoriasis. The present study was designed to analyze the effect of narrowband ultraviolet B therapy on these proteins.

*S100A8*, *S100A9* gene expression and S100A8/A9 heterocomplex protein levels were analyzed in lesional and non-lesional skin before and after narrowband-UVB treatment in patients with chronic plaque type psoriasis. In addition, disease severity was measured by psoriasis area and severity index (PASI) and serum protein levels of S100A8/A9 were repeatedly analyzed. Narrowband-UVB treatment significantly reduced *S100A8*, *S100A9* gene expression and S100A8/A9 protein levels in lesional skin while serum levels showed no significant change. No correlation between PASI and serum S100A8/A9 protein levels was found. These results implicate a role of S100A8/A9 in the anti-inflammatory effect of narrowband-UVB. Serum S100A8/A9 levels do not respond to treatment suggesting that serum S100A8/A9 does not originate from psoriasis skin keratinocytes. Serum S100A8/A9 levels do not correlate with PASI questioning serum S100A8/A9 as a biomarker for psoriasis skin activity.

Trial Registration: DRKS 00014817.

## Introduction

The S100A8/S100A9 heterocomplex (calprotectin) is composed of S100A8 and S100A9 proteins (also termed MRP8 and MRP14) and are subgroups of the S100 calgranulin family [1, 2]. The genes coding for *S100A8* and *S100A9* are located within the psoriasis susceptibility locus 4 (PSORS4) mapped to chromosome 1q21. S100A8 and S100A9 are multifunctional proteins, and it is hypothesized that S100A8 and S100A9 functions vary with concentration and oxidation of the S100A8/A9 heterocomplex. S100A8/A9 properties include oxidant scavenging, antimicrobial activity, leukocyte chemoattractant and chemokine-like activities suggesting a regulatory role in inflammation [3, 4]. S100A8 and S100A9 proteins are primarily expressed in neutrophils where they compose 40% of the cytosolic content and in monocytes/macrophages [5]. Keratinocytes express S100A8/A9 in response to stress, including wound healing, tape stripping, and exposure to detergents [6].

S100A8/A9 has become a widespread biomarker used in the diagnosis and monitoring of inflammatory bowel disease (IBD) [7] and has been proposed as a potential biomarker for psoriasis and psoriasis arthritis (PsA) [8, 9]. S100A8/A9 is present at high levels in extracellular fluids (synovia, blood and sputum) in various inflammatory diseases such as rheumatoid arthritis and cystic fibrosis [3, 5]. Serum levels of S100A8/A9 in middle age healthy individuals correlate to blood neutrophil counts [10] and high levels of circulating S100A8/A9 are associated with increased risk for cardiovascular events and carotid arteriosclerosis [11]. Plasma S100A8/A9 has a short half-life of approximately 5 hours [12].

In psoriasis mouse models, deletion of S100A9 improves psoriasis-like skin disease, and studies on adenovirus induced overexpression of S100A8/A9 in human keratinocytes show increased levels of TNF-alpha, IL-6 and IL-8 in cell medium suggesting a pro-inflammatory effect and important role for S100A8/A9 in psoriasis pathogenesis [13, 14].

*S100A8* and *S100A9* are exceedingly upregulated in the epidermis in lesional skin of patients with psoriasis [13] and histopathological analysis of psoriatic lesions show increased levels of S100A8/A9 in keratinocytes compared to healthy skin [15–17]. These findings led to the assumption that S100A8 and S100A9 might be potential therapeutic targets for the treatment of psoriasis [15]. Previous genomic transcriptome studies on the effect of NB-UVB in psoriasis showed downregulation of *S100A8* and *S100A9* in response to NB-UVB [18, 19]. However, these studies included only 3 and 11 patients and did not analyse protein expression. Benoit et al. revealed increased S100A8/A9 protein serum levels in patients with psoriasis compared to healthy controls as well as a positive correlation with disease severity measured by PASI [8]. The authors suggested keratinocytes from lesional skin to be the source of elevated serum S100A8/A9 levels. However, other studies have not been able to confirm a correlation between disease severity and S100A8/A9 serum levels [9, 20, 21] but proposed that S100A8/A9 might be a potential biomarker of PsA [9, 20].

Only scarce data is available on the response of S100A8/A9 protein expression to psoriasis treatment. One previous study showed that etanercept treatment reduces S100A8/A9 protein levels in serum [21].

The present study was designed to analyse skin and serum expression of S100A8/A9 before and after NB-UVB treatment in patients with chronic plaque type psoriasis. The correlation between psoriasis area and severity index (PASI) and serum levels was studied to investigate a potential role of serum S100A8/A9 as a biomarker for skin disease severity.

## Materials and methods

### Study subjects

Patients with chronic plaque psoriasis eligible for NB-UVB treatment were recruited for the study. According to the national treatment recommendations of the Swedish Society of Dermatology and Venereology NB-UVB is primarily offered to plaque psoriasis patients with a PASI value of 3–9 where topical treatment is insufficient to obtain disease control. All participants were included at the dermatology outpatient clinic at Ryhov hospital, Jönköping, Sweden (December 2013 to March 2016). Exclusion criteria were ongoing systemic anti-inflammatory treatment (including any systemic treatment for psoriasis), PsA or other rheumatological diseases, IBD, other inflammatory dermatoses, pregnancy, intense UV exposure two weeks prior to study start (i.e. vacation, tanning salon), chronic infectious diseases, exposure to photosensitizing drugs and age < 18 years.

The study was conducted in compliance with good clinical practice and according to the Declaration of Helsinki Principles. All subjects received written study information prior to recruitment and written informed consent was obtained. Study protocols were approved by the ethical committee at Linköping University, Linköping, Sweden (Dnr. 2012/428-31) (S10.1 and S10.2). Age, sex, body mass index (BMI) and current medication were recorded for all patients. A minimum sample size of n = 25 was determined based on the assumption that NB-UVB would have an effect size corresponding to the magnitude of the difference of serum S100A8/A9 levels from patients with psoriasis selected at random in the dermatology department (n = 47) and healthy blood donors (n = 8) at Ryhov hospital, Jönköping, Sweden. Power (1-β) being set to 0.8 and α to 0.05 (S1 Sample size). Out of 30 recruited patients, 27 completed a full NB-UVB treatment (21.4±4.2 NB-UVB sessions). Dropouts were due to severe illness, change of residence and unspecified personal reason. Dropout average baseline disease severity was similar to subjects who fulfilled the study (PASI 7.6 vs PASI 7.9).

### Study design

NB-UVB (311nm) therapy was administered using a Waldmann 7002 cabin (Waldmann Medizintechnik, Villingen-Schwenningen, Germany). Patients were treated 2.3 times (±0.7) per week and the mean treatment period was 10.4 weeks (±3.6). The mean maximum dose reached was 2.64 J/cm2 (±1.2) at the end of the treatment period. Energy output was measured with a standard intrinsic UV meter. Initial dose was dependent on skin phototype and increased 20% at each visit if well tolerated. When a previous treatment resulted in erythema, the dose was maintained or the dose was decreased, depending on whether the erythema was asymptomatic or severe and painful.

**Fig 1:**
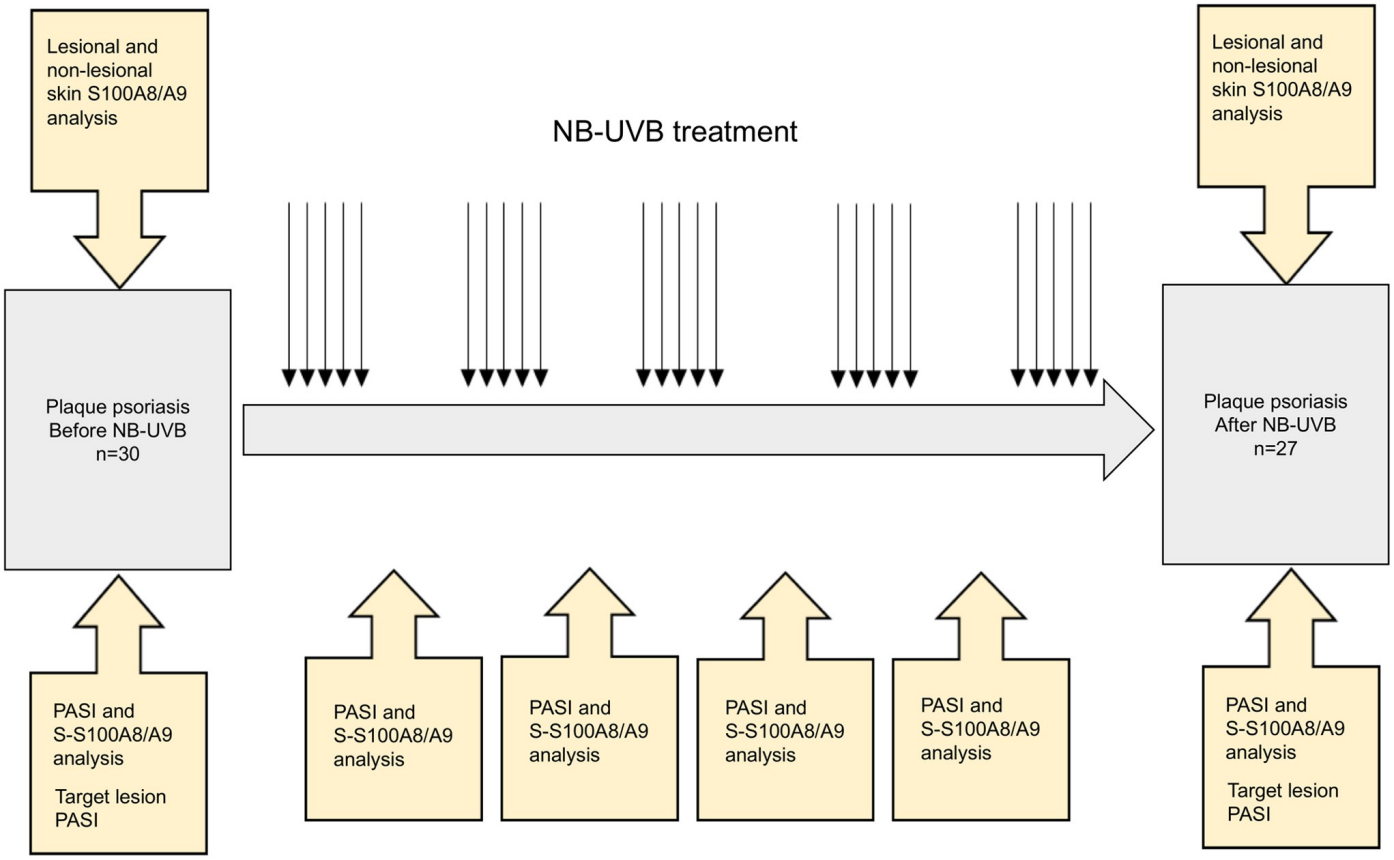
Schematic figure of the study protocol. Arrows represent NB-UVB treatment sessions.

Serum S100A8/A9 protein and PASI levels were measured before the first, 6th, 11th, 16th, 21st, 26th and final treatment. The exact number of treatment sessions was not predetermined by the study design but based on clinical response and motivation of the study subjects to continue treatment. The NB-UVB regimen was designed to be as similar to the ordinary clinical setting as possible. Before the first and the final NB-UVB treatment session two 2 mm skin biopsies from the central parts of a single psoriasis plaque (target lesion) and two 2 mm skin biopsies from non-lesional skin 10 cm from target lesion site were taken for S100A8/A9 gene and protein analysis (Fig 1). Target lesions were selected on the basis of being representative of the patients general disease (assessed by dermatologists AD, MA and OS), being distinct as to be easily identified in follow up, not located on face, chest, distal parts of arms and hands to avoid scars in these locations. Target lesion psoriasis severity and total body PASI were recorded before biopsy sampling. PASI was first introduced by Fredriksson and Pettersson in 1978 and is currently a widespread method for disease evaluation [22, 23]. Target lesion severity was calculated by adding PASI scores (0–4) for erythema, induration and desquamation of the target lesion and was denoted “target PASI”. Participants were instructed not to treat the target lesion with topical treatment other than moisturizing cream. For other lesions, topical treatment was restricted to moisturizing cream and mometason 0.1%. The latter was to be applied to a maximum of 30% of the total body surface area.

#### S100A8/A9 protein analysis

Venous blood samples for protein analysis were collected in BD Vacutainer gel tubes (Becton, Dickinson and Company). After 30 minutes the tubes were centrifuged at 2000xg for 10 minutes at room temperature. 300 μl serum was frozen at -80°C for subsequent protein analysis for each sample.

Skin biopsies sampled for protein analysis were immediately stored at -80°C. Before protein analysis, samples were homogenized in RIPA Lysis Buffer System (Santa Cruz Biotechnology) using Tissue Lyser II (Qiagen) set at 3 + 3 minutes, 30Hz using 7 mm stainless beads. Serum and skin levels of S100A8/A9 heterocomplex were analysed using Phadia EliA Calprotectin assay (Thermo Fisher Scientific).

#### S100A8/A9 RNA purification and gene expression analysis

Skin biopsies sampled for gene expression analysis were immediately immersed in RNA*later* RNA Stabilization Reagent (Qiagen) and stored at 4°C. After 24 hours RNA*later* was removed and samples were frozen at -196 °C.

Total RNA was purified according to manufacturer’s instructions. Skin biopsies were homogenized using a TissueRuptor and disposable probes (Qiagen). RNA was purified using the RNeasy Fibrous Tissues mini kit (Qiagen). Concentration and purity was measured using Nanodrop ND-1000 (Thermo Fisher Scientific Inc.), and RNA integrity was assessed with the 2100 Bioanalyzer (Agilent technologies).

RNA reverse transcription was performed using the High capacity cDNA reverse transcription kit with RNase inhibitor (Applied Biosystems), according to the manufacturer’s instructions, and resulting cDNA was stored at -80 °C.

Gene expression was analyzed on the 7500 Fast real-time PCR system (Applied Biosystems) and the standard run mode using Taq-Man Universal Master Mix no UNG (Applied Biosystems) and Taqman Gene Expression Assays (Applied Biosystems) for *S100A8* (Hs00374264_g1), *S100A9* (Hs00610058_m1), *TBP* (Hs00427620_m1), *ACTB* (Hs99999903_m1) and *GAPDH* (Hs03929097_g1). For each assay and sample, cDNA based on 10 ng total RNA were analyzed in a total volume of 20 μl.

Threshold cycle (Ct) values were established using the 7500 software (version 2.0.6, Applied Biosystems). Reference genes (*TBP*, *ACTB* and *GAPDH*) were evaluated for low sample-to-sample variation using the NormFinder [24] algorithm implemented in GenEx Professional software (version 5.4.2.128, MultiD Analyses AB). Ct values were normalized to the *TBP* reference gene showing the best stability value and GenEx Professional software was used to compute relative gene expression based on the comparative Ct (2^-ΔΔct^) method [25].

### Statistics

Results are expressed in tables as mean ± standard deviation (SD). The Shapiro-Wilk test was used to test for normality (S1 Table) and Wilcoxon signed rank test to assess the effect of NB-UVB on S100A8/A9 levels. Kendall´s tau rank correlation (correlation coefficient τ) was used to measure non-parametric ordinal association. Statistical analyses and graphical presentation were performed using IBM SPSS statistics (Version 22) and GraphPad Prism (Version 7.0b). G*Power (Version 3.1.9.3) was used to calculate sample size. P<0.05 was considered statistically significant. Dropout subjects were not included in statistical analysis.

## Results

### Treatment response

Out of 30 recruited study participants, 27 fulfilled NB-UVB treatment as described in the method section (Fig 1). 82% of patients reached PASI50 (at least 50% improvement of PASI) and 52% PASI75. Demographics of study population (age, gender, BMI) and details of treatment regimen, duration, and outcome are summarized in Table 1.

**Table 1:** Patients’ demographic data and NB-UVB treatment outcomes.

Number of subjects	27
Number of NB-UVB sessions	21.4 (4.2)*
Age	48.2 (14.6)*
Male: female ratio	21:6
BMI	26.7 (4.2)*
PASI before treatment	7.9 (4.6)*
PASI after treatment	2.1 (1.8)*
Average PASI improvement	69.6% (22.7)*
Percentage achieving PASI75	52%
Target plaque PASI before treatment	5.2 (1.4)*
Target plaque PASI after treatment	1.1 (1.3)*
Average target plaque PASI improvement	76.8% (29.5)*
Percentage achieving target plaque PASI75	74%

*Data shown as mean with standard deviation (SD).

#### Increased S100A8/A9 expression in lesional skin compared to non-lesional skin

**Fig 2:**
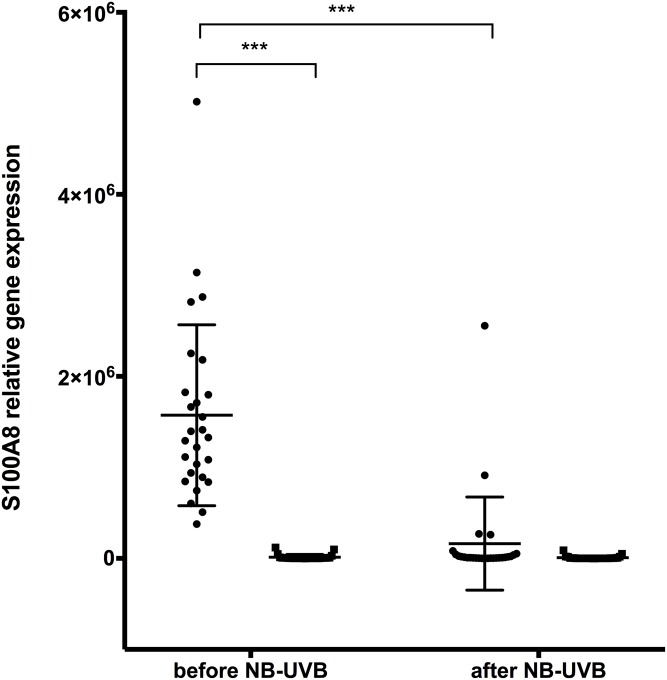
Relative S100A8 gene expression in lesional and non-lesional skin of patients with psoriasis before and after NB-UVB treatment. n = 27, ● = lesional skin, ■ = non-lesional skin. (*** p<0.001).

**Fig 3:**
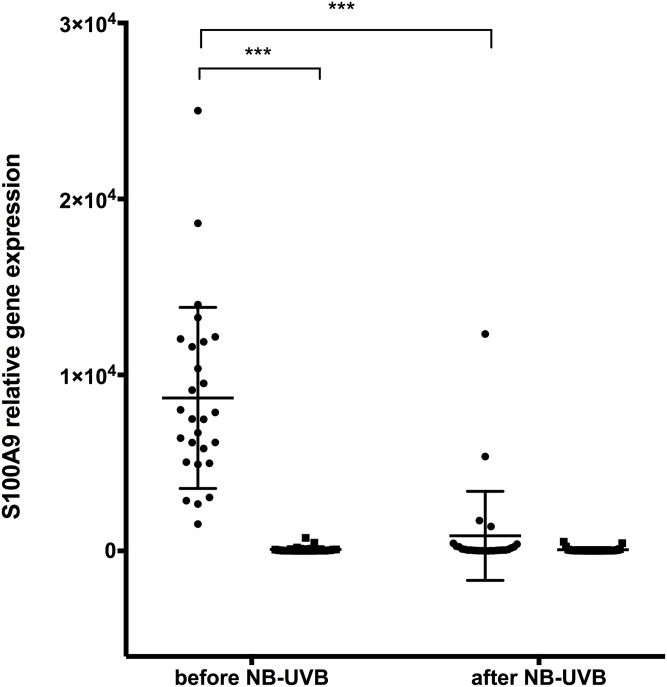
Relative gene expression of S100A9 in lesional and non-lesional skin of patients with psoriasis before and after NB-UVB treatment. n = 27, ● = lesional skin, ■ = non-lesional skin. (*** p<0.001).

**Fig 4:**
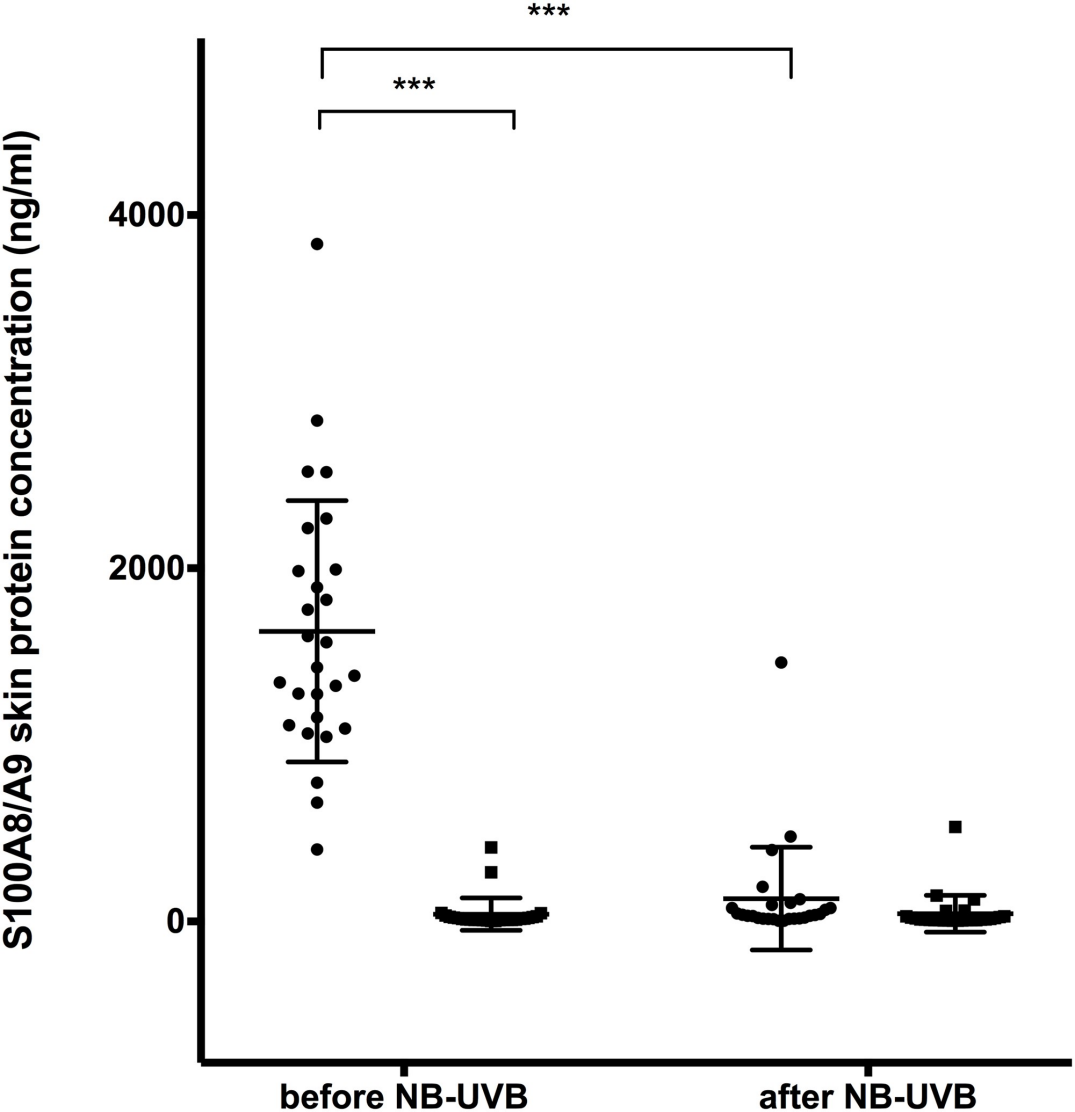
Skin protein levels of S100A8/A9 heterocomplex in lesional and non-lesional skin of patients with psoriasis before and after NB-UVB treatment. n = 27, ● = lesional skin, ■ = non-lesional skin. (*** p<0.001).

*S100A8* and *S100A9* gene expression in lesional skin was significantly elevated compared to non-lesional skin before start of NB-UVB treatment (Figs 2 and 3). Results from protein analysis were consistent with gene expression results showing significantly increased S100A8/A9 protein levels in lesional skin compared to non-lesional skin before NB-UVB therapy (Fig 4).

#### S100A8/A9 expression is significantly decreased in lesional skin after NB-UVB treatment

After NB-UVB treatment, skin biopsies from lesional skin showed significantly reduced *S100A8* and *S100A9* gene expression compared to levels before NB-UVB treatment (Figs 2 and 3). Gene expression results were consistent with skin protein expression of S100A8/A9, which also showed a significant decrease after NB-UVB (Fig 4).

#### Correlation of S100A8 and S100A9 gene expression before and after NB-UVB

Gene expression of *S100A8* and of *S100A9* are significantly correlated in lesional skin (τ_b_ = 0.823, p<0.01 and τ_b_ = 0.863, p<0.01) and in non-lesional skin (τ_b_ = 0.778, p<0.01 and τ_b_ = 0,715, p<0.01) before and after NB-UVB.

#### No significant effect of NB-UVB treatment on serum S100A8/A9 levels

**Fig 5:**
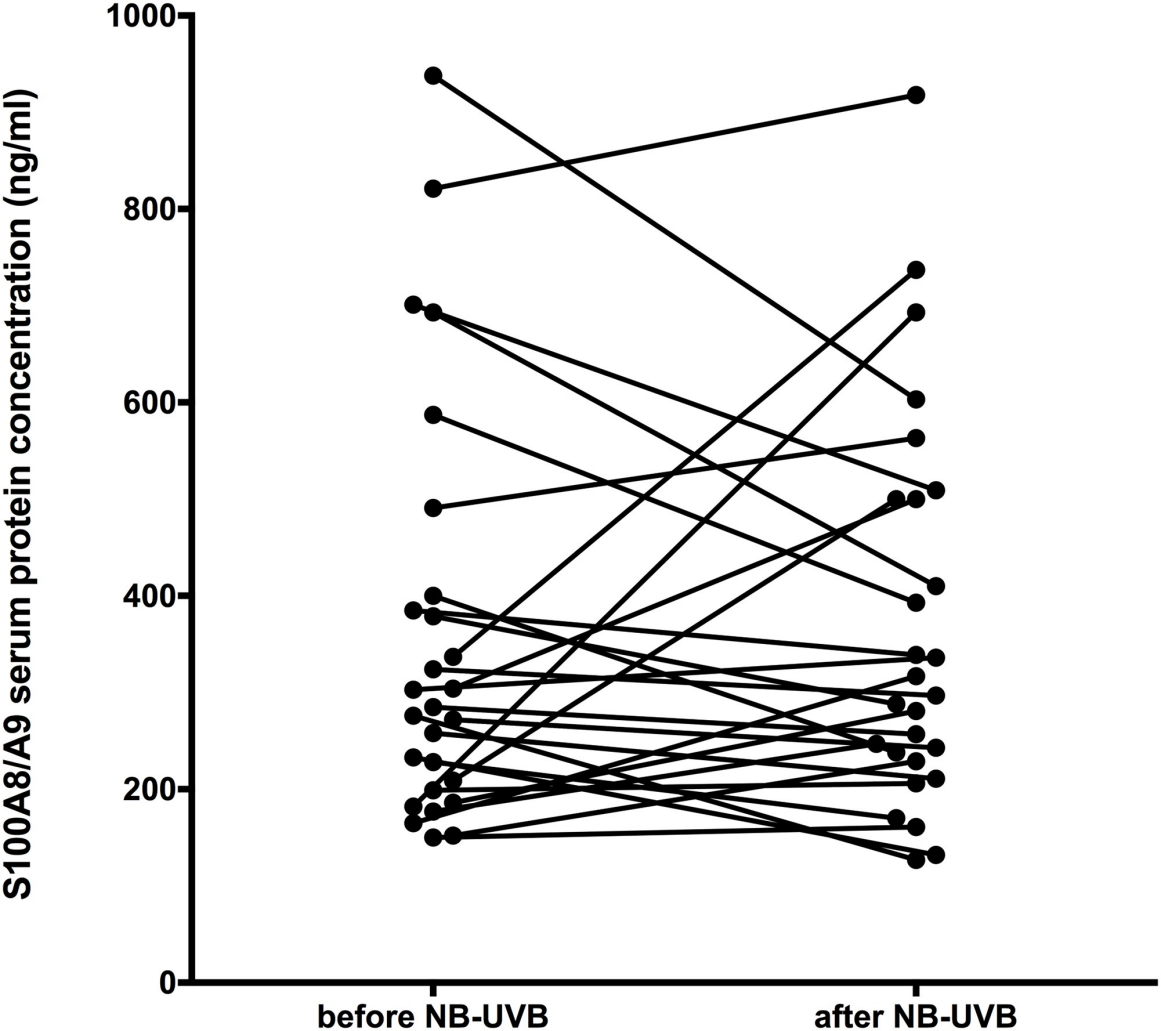
Serum S100A8/A9 levels before and after completion of NB-UVB psoriasis treatment. No significant difference was found (n = 27).

Serum protein levels of S100A8/A9 heterocomplex before (357ng/ml ± 214) and after (369ng/ml ± 200) NB-UVB treatment showed no significant difference (Fig 5). The significant decrease of S100A8/A9 observed in skin levels was not followed by a reduction of S100A8/A9 serum levels.

#### No correlation between PASI and serum S100A8/A9

**Fig 6:**
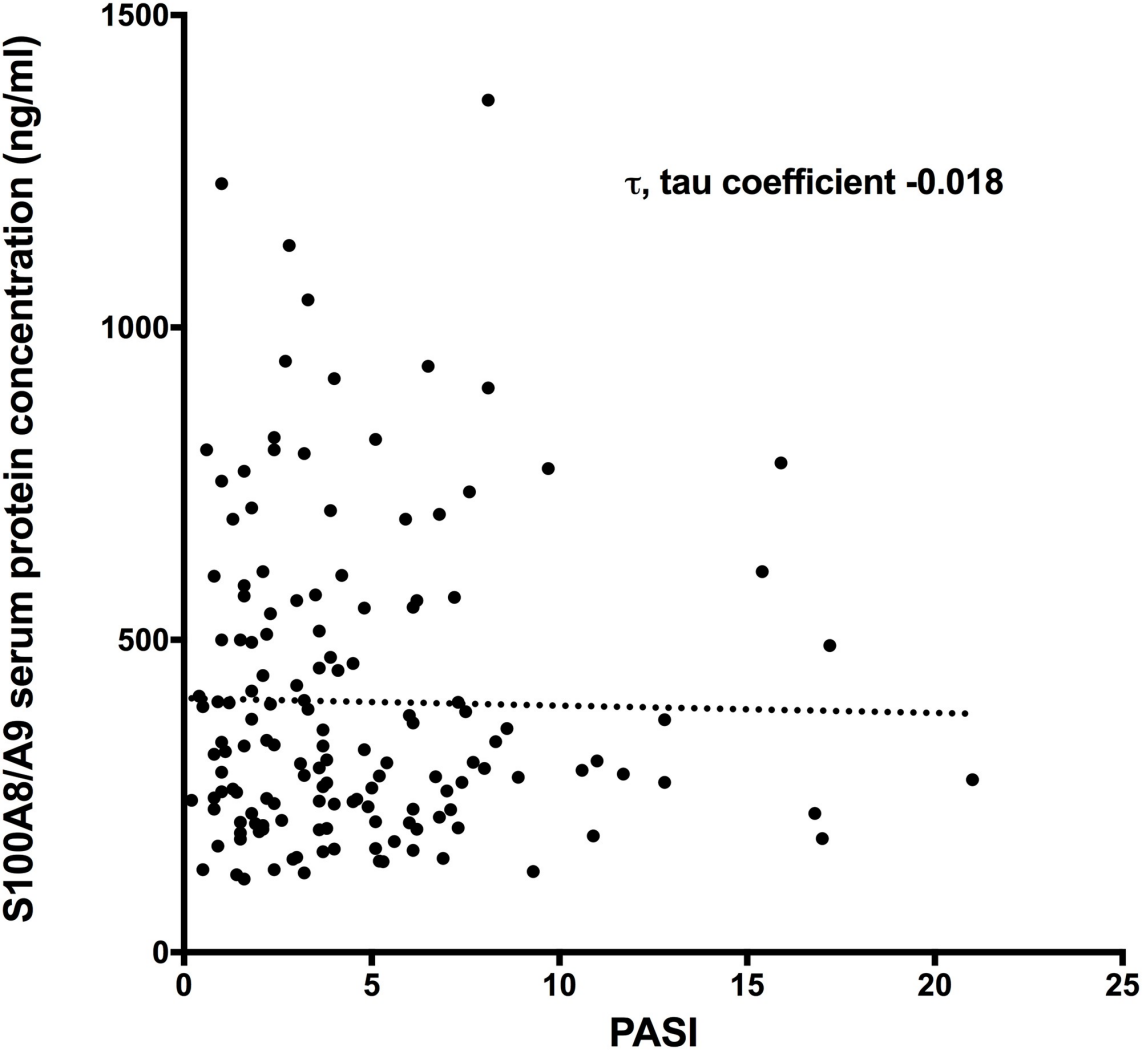
Psoriasis severity (PASI) and serum S100A8/A9 protein levels before, during and after NB-UVB treatment. No correlation was found between PASI and serum S100A8/A9 protein.

Monitoring PASI and serum S100A8/A9 levels during NB-UVB treatment (Fig 1) resulted in 145 specific PASI and serum S100A8/A9 measurements (Fig 6). No correlation between PASI levels and serum S100A8/A9 levels was found (correlation coefficient, τ -0.018).

#### Correlation of S100A8/A9 levels in lesional skin and target lesion PASI

**Fig 7:**
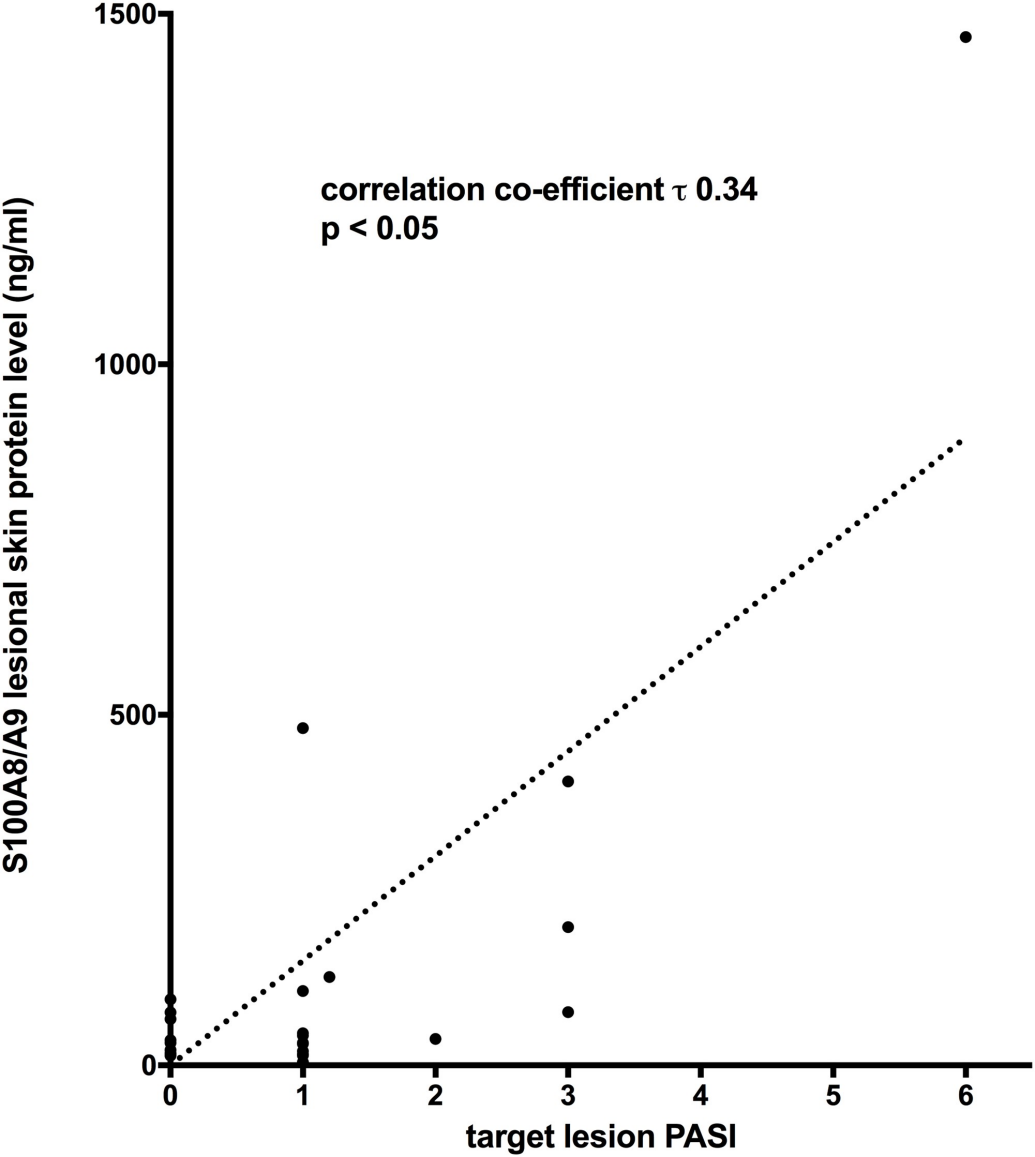
S100A8/A9 protein in lesional skin after NB-UVB and target lesion PASI. A moderate correlation was found.

Protein levels of S100A8/A9 showed a moderate correlation with target lesion PASI after NB-UVB (correlation coefficient τ 0.34 p< 0.05) (Fig 7) but not before NB-UVB (S1 Fig).

## Discussion

Several S100 proteins and particularly S100A8 and S100A9 are highly upregulated in psoriasis plaques [6, 26]. Hence, potential roles for S100A8 and S100A9 both as treatment targets and biomarkers for psoriasis disease severity have been proposed.

The present study shows for the first time that NB-UVB treatment significantly suppress elevated S100A8/A9 protein heterocomplex levels in lesional skin to levels comparable to those found in non-lesional skin. These findings are supported by corresponding changes in skin gene expression. Interestingly, our results show that treatment with NB-UVB has no significant effect on serum levels of S100A8/A9. These results suggest that elevated serum S100A8/A9 levels in individuals with psoriasis do not originate from lesional skin keratinocytes as previously suggested by Benoit et al. [8]. As proposed in earlier studies on S100A8/A9 in healthy middle-aged individuals and patients with systemic lupus erythematosus the origin of serum S100A8/A9 could be circulating neutrophils or thrombocytes. Elevated S100A8/A9 levels in these studies were associated with increased risk for cardiovascular disease [10, 27] implicating that increased S100A8/A9 serum concentrations could be a marker of systemic inflammation rather than skin inflammation in individuals with psoriasis. Since etanercept treatment induces significant serum S100A8/A9 reduction in patients with psoriasis [21] S100A8/A9 might be involved in the systemic anti-inflammatory effect of biologics. The expression of *S100A15* (Koebnerisin) and *S100A7* (Psoriasin) are increased in circulating peripheral blood mononuclear cells (PBMCs) in patients with psoriasis [28]. If S100A8 and S100A9 share similar expression patterns then it is possible that PBMCs could be a source of elevated S100A8/A9 in serum.

NB-UVB is thought to exert immunosuppression in psoriasis partly by inhibiting Th17 and interferon signalling pathways in the skin [18]. It is not fully elucidated if NB-UVB therapy has effect on systemic inflammation in psoriasis. Some studies have shown decreased levels of *IL-17A*, *TNF-α*, *S100A15*, *S100A7*, *S100A9* and *IL-6* gene expression in PBMCs after NB-UVB treatment in patients with psoriasis [28, 29] whereas other pro-inflammatory mediators such as sVCAM-1, sICAM-1, sE-selectin, MMP-9, and MPO showed no significant reduction [30]. The latter proposing an anti-inflammatory effect of NB-UVB solely restricted to the skin which is consistent with our data showing no effect of NB-UVB on serum S100A8/A9 levels.

Only scarce data is available describing the effect of NB-UVB treatment on the expression of S100A8/A9 in skin from patients with psoriasis. One previous study showed that NB-UVB downregulates *S100A9* gene expression in psoriasis skin and another revealed downregulation of *S100A8*. Both studies were performed on small samples, including 11 and 3 patients [18, 19]. Acute UV radiation induces in vivo upregulation of *S100A8* in the skin [31] whereas UVA radiation induces S100A8, but not S100A9 in murine epidermis and keratinocyte cell lines possibly by oxidative stress [32]. It is completely unknown how UVB interferes with S100A8/A9 expression but it is interesting to speculate that the anti-inflammatory effect of NB-UVB treatment might partly be mediated by the reduction of S100A8/A9 expression in lesional skin.

Our data show no correlation between PASI and serum S100A8/A9 concentration before, during or after UVB treatment suggesting serum S100A8/A9 to be unsuitable to quantify skin psoriasis disease activity. Corresponding results were obtained in a recent study showing that S100A7 and S100A12 serum levels correlate with disease severity whereas S100A8/A9 does not [21]. However, previous studies on serum S100A8/A9 concentration and PASI have shown contradictory results. Hansson et al found no correlation in a study including 65 patients with psoriasis and PsA [9] while Benoit et al showed a significant correlation between PASI and S100A8/A9 serum concentration [8]. Naik et al found a correlation between S100A8/A9 levels, vascular inflammation and PASI [33]. However, this study included patients with systemic treatment which may have affected serum S100A8/A8 levels. Due to inconsistent results in these previous studies the final role for S100A8/A9 as a psoriasis biomarker cannot be defined. Larger studies are needed to further investigate the correlation between skin inflammation, PASI and serum S100A8/A9 concentration. Notably, our results show that S100A8/A9 plaque protein levels correlate with target plaque PASI after NB-UVB but not before treatment. Target PASI in itself is a blunt scale and might not be sufficient to accurately describe the severity of a single plaque.

Limitations in the present study lie in the inclusion of patients with mainly low PASI scores (22 out of 27 had PASI ≤10). Patients with more extensive and severe skin disease might show higher levels of S100A8/A9 and needs to be investigated. The number of patients included in our study may have been too small assuming that the effect of NB-UVB on serum S100A8/A9 was overestimated in our sample size calculation. However, our data revealed no trend towards serum reduction despite the substantial changes seen in lesional skin. The present sample size limited further subgroup analyses and did not allow comparison neither of non-responders versus responders nor severe disease versus moderate disease. NB-UVB treatment intervention in the current study followed the standard clinical setting and in this regard, other confounding factors biasing the results cannot be completely excluded. The patients included did not use systemic drugs for psoriasis and were not allowed to have systemic inflammatory diseases such as arthritis, IBD or chronic infections. However, it cannot be completely ruled out that other diseases (e.g. hypertension, hyperlipidemia) and treatments could have affected the present results. We decided not to include a control group since the present study was conducted to analyze differences between lesional and non-lesional skin and since NB-UVB treatment theoretically could increases the risk of skin cancer in healthy individuals.

In conclusion, the current study shows for the first time a significant reduction in S100A8/A9 skin protein levels after NB-UVB treatment while serum S100A8/A9 protein concentration remained unaffected. These results suggest that skin keratinocytes may not be the major source of elevated circulating S100A8/A9 in patients with psoriasis as previously suggested by Benoit [8]. Further studies are required to identify the source of elevated serum S100A8/A9 levels, which could lead to a better understanding of systemic inflammation and comorbidities in psoriasis. The present study shows no correlation between PASI and serum S100A8/A9 concentration questioning serum S100A8/A9 as biomarker for psoriasis skin disease activity.

## Supporting information

S1 Sample size Calculation of sample size. (PDF)

S1 Table Results of normality tests. (PDF)

S1 Fig Plotting S100A8/A9 protein in lesional skin before NB-UVB shows no correlation with target lesion skin disease activity expressed as target lesion PASI. (TIFF)

S1 Dataset Raw data, skin S100A8/A9 protein levels. (XLSX)

S2 Dataset Raw data, serum S100A8/A9 protein levels. (XLSX)

S3 Dataset Raw data, S100A8, S100A9 and reference gene expression. (XLSX)

S4 Dataset Raw data, study subjects background data and PASI scores. (XLSX)

S1 TREND checklist File containing TREND checklist. (PDF)

S1 Text Study protocol for ethical board. (DOC)

S2 Text Modifications to original study protocol. (DOCX)

## References

[1] Pruenster M, Vogl T, Roth J, Sperandio M. S100A8/A9: From basic science to clinical application. Pharmacol Ther. 2016;167:120–31. 10.1016/j.pharmthera.2016.07.015 27492899

[2] Foell D, Wittkowski H, Vogl T, Roth J. S100 proteins expressed in phagocytes: a novel group of damage-associated molecular pattern molecules. J Leukoc Biol. 2007;81(1):28–37. 10.1189/jlb.0306170 16943388

[3] Yui S, Nakatani Y, Mikami M. Calprotectin (S100A8/S100A9), an inflammatory protein complex from neutrophils with a broad apoptosis-inducing activity. Biol Pharm Bull. 2003;26(6):753–60. 12808281 10.1248/bpb.26.753

[4] Hsu K, Champaiboon C, Guenther BD, Sorenson BS, Khammanivong A, Ross KF, et al Anti-Infective Protective Properties of S100 Calgranulins. Antiinflamm Antiallergy Agents Med Chem. 2009;8(4):290–305. 20523765 10.2174/187152309789838975 PMC2879674

[5] Perera C, McNeil HP, Geczy CL. S100 Calgranulins in inflammatory arthritis. Immunol Cell Biol. 2010;88(1):41–9. 10.1038/icb.2009.88 19935766

[6] Eckert RL, Broome AM, Ruse M, Robinson N, Ryan D, Lee K. S100 proteins in the epidermis. J Invest Dermatol. 2004;123(1):23–33. 10.1111/j.0022-202X.2004.22719.x 15191538

[7] Leach ST, Yang Z, Messina I, Song C, Geczy CL, Cunningham AM, et al Serum and mucosal S100 proteins, calprotectin (S100A8/S100A9) and S100A12, are elevated at diagnosis in children with inflammatory bowel disease. Scand J Gastroenterol. 2007;42(11):1321–31. 10.1080/00365520701416709 17852869

[8] Benoit S, Toksoy A, Ahlmann M, Schmidt M, Sunderkotter C, Foell D, et al Elevated serum levels of calcium-binding S100 proteins A8 and A9 reflect disease activity and abnormal differentiation of keratinocytes in psoriasis. Br J Dermatol. 2006;155(1):62–6. 10.1111/j.1365-2133.2006.07198.x 16792753

[9] Hansson C, Eriksson C, Alenius GM. S-calprotectin (S100A8/S100A9): a potential marker of inflammation in patients with psoriatic arthritis. J Immunol Res. 2014;2014:696415 10.1155/2014/696415 24955375 PMC4053083

[10] Cotoi OS, Duner P, Ko N, Hedblad B, Nilsson J, Bjorkbacka H, et al Plasma S100A8/A9 correlates with blood neutrophil counts, traditional risk factors, and cardiovascular disease in middle-aged healthy individuals. Arterioscler Thromb Vasc Biol. 2014;34(1):202–10. 10.1161/ATVBAHA.113.302432 24202303

[11] Schiopu A, Cotoi OS. S100A8 and S100A9: DAMPs at the crossroads between innate immunity, traditional risk factors, and cardiovascular disease. Mediators Inflamm. 2013;2013:828354 10.1155/2013/828354 24453429 PMC3881579

[12] Fagerhol MK, Nielsen HG, Vetlesen A, Sandvik K, Lyberg T. Increase in plasma calprotectin during long-distance running. Scand J Clin Lab Invest. 2005;65(3):211–20. 16095050 10.1080/00365510510013587

[13] Schonthaler HB, Guinea-Viniegra J, Wculek SK, Ruppen I, Ximenez-Embun P, Guio-Carrion A, et al S100A8-S100A9 protein complex mediates psoriasis by regulating the expression of complement factor C3. Immunity. 2013;39(6):1171–81. 10.1016/j.immuni.2013.11.011 24332034

[14] Lee Y, Jang S, Min JK, Lee K, Sohn KC, Lim JS, et al S100A8 and S100A9 are messengers in the crosstalk between epidermis and dermis modulating a psoriatic milieu in human skin. Biochemical and biophysical research communications. 2012;423(4):647–53. 10.1016/j.bbrc.2012.05.162 22683330

[15] Chimenti MS, Triggianese P, Botti E, Narcisi A, Conigliaro P, Giunta A, et al S100A8/A9 in psoriatic plaques from patients with psoriatic arthritis. J Int Med Res. 2016;44(1 suppl):33–7. 10.1177/0300060515598900 27683136 PMC5536520

[16] Liu H, Huang K, Wu Y, Lin N, Li J, Tu Y. The expression of interleukin-22 and S100A7, A8, A9 mRNA in patients with psoriasis vulgaris. J Huazhong Univ Sci Technolog Med Sci. 2007;27(5):605–7. 10.1007/s11596-007-0533-z 18060647

[17] Park CC, Kim KJ, Woo SY, Chun JH, Lee KH. Comparison of the Expression Profile of JunB, c-Jun, and S100A8 (Calgranulin A) in Psoriasis Vulgaris and Guttate Psoriasis. Ann Dermatol. 2009;21(1):35–8. 10.5021/ad.2009.21.1.35 20548852 PMC2883365

[18] Racz E, Prens EP, Kurek D, Kant M, de Ridder D, Mourits S, et al Effective treatment of psoriasis with narrow-band UVB phototherapy is linked to suppression of the IFN and Th17 pathways. J Invest Dermatol. 2011;131(7):1547–58. 10.1038/jid.2011.53 21412260

[19] Hochberg M, Zeligson S, Amariglio N, Rechavi G, Ingber A, Enk CD. Genomic-scale analysis of psoriatic skin reveals differentially expressed insulin-like growth factor-binding protein-7 after phototherapy. Br J Dermatol. 2007;156(2):289–300. 10.1111/j.1365-2133.2006.07628.x 17223869

[20] Aochi S, Tsuji K, Sakaguchi M, Huh N, Tsuda T, Yamanishi K, et al Markedly elevated serum levels of calcium-binding S100A8/A9 proteins in psoriatic arthritis are due to activated monocytes/macrophages. J Am Acad Dermatol. 2011;64(5):879–87. 10.1016/j.jaad.2010.02.049 21315480

[21] Wilsmann-Theis D, Wagenpfeil J, Holzinger D, Roth J, Koch S, Schnautz S, et al Among the S100 proteins, S100A12 is the most significant marker for psoriasis disease activity. J Eur Acad Dermatol Venereol. 2016;30(7):1165–70. 10.1111/jdv.13269 26333514

[22] Fredriksson T, Pettersson U. Severe psoriasis—oral therapy with a new retinoid. Dermatologica. 1978;157(4):238–44. 357213 10.1159/000250839

[23] Spuls PI, Lecluse LL, Poulsen ML, Bos JD, Stern RS, Nijsten T. How good are clinical severity and outcome measures for psoriasis?: quantitative evaluation in a systematic review. J Invest Dermatol. 2010;130(4):933–43. 10.1038/jid.2009.391 20043014

[24] Andersen CL, Jensen JL, Orntoft TF. Normalization of real-time quantitative reverse transcription-PCR data: a model-based variance estimation approach to identify genes suited for normalization, applied to bladder and colon cancer data sets. Cancer research. 2004;64(15):5245–50. 10.1158/0008-5472.CAN-04-0496 15289330

[25] Livak KJ, Schmittgen TD. Analysis of relative gene expression data using real-time quantitative PCR and the 2(-Delta Delta C(T)) Method. Methods. 2001;25(4):402–8. 10.1006/meth.2001.1262 11846609

[26] Broome AM, Ryan D, Eckert RL. S100 protein subcellular localization during epidermal differentiation and psoriasis. J Histochem Cytochem. 2003;51(5):675–85. 10.1177/002215540305100513 12704215 PMC3785113

[27] Lood C, Tyden H, Gullstrand B, Jonsen A, Kallberg E, Morgelin M, et al Platelet-Derived S100A8/A9 and Cardiovascular Disease in Systemic Lupus Erythematosus. Arthritis Rheumatol. 2016;68(8):1970–80. 10.1002/art.39656 26946461

[28] Batycka-Baran A, Hattinger E, Zwicker S, Summer B, Zack Howard OM, Thomas P, et al Leukocyte-derived koebnerisin (S100A15) and psoriasin (S100A7) are systemic mediators of inflammation in psoriasis. J Dermatol Sci. 2015;79(3):214–21. 10.1016/j.jdermsci.2015.05.007 26055798

[29] Batycka-Baran A, Besgen P, Wolf R, Szepietowski JC, Prinz JC. The effect of phototherapy on systemic inflammatory process in patients with plaque psoriasis. J Photochem Photobiol B. 2016;161:396–401. 10.1016/j.jphotobiol.2016.05.023 27314537

[30] Sigurdardottir G, Ekman AK, Stahle M, Bivik C, Enerback C. Systemic treatment and narrowband ultraviolet B differentially affect cardiovascular risk markers in psoriasis. J Am Acad Dermatol. 2014;70(6):1067–75. 10.1016/j.jaad.2013.12.044 24656729

[31] Lee YM, Kim YK, Eun HC, Chung JH. Changes in S100A8 expression in UV-irradiated and aged human skin in vivo. Arch Dermatol Res. 2009;301(7):523–9. 10.1007/s00403-009-0960-8 19466434

[32] Grimbaldeston MA, Geczy CL, Tedla N, Finlay-Jones JJ, Hart PH. S100A8 induction in keratinocytes by ultraviolet A irradiation is dependent on reactive oxygen intermediates. J Invest Dermatol. 2003;121(5):1168–74. 10.1046/j.1523-1747.2003.12561.x 14708622

[33] Naik HB, Natarajan B, Stansky E, Ahlman MA, Teague H, Salahuddin T, et al Severity of Psoriasis Associates With Aortic Vascular Inflammation Detected by FDG PET/CT and Neutrophil Activation in a Prospective Observational Study. Arterioscler Thromb Vasc Biol. 2015;35(12):2667–76. 10.1161/ATVBAHA.115.306460 26449753 PMC4662627

