# Supplementary material for: Observational study on Swedish plaque psoriasis patients receiving narrowband-UVB treatment show decreased S100A8/A9 protein and gene expression levels in lesional psoriasis skin but no effect on S100A8/A9 protein levels in serum
Source: PLoS One. 2019 Mar 13;14(3):e0213344. doi: 10.1371/journal.pone.0213344 (PMC6415841; doi:10.1371/journal.pone.0213344)
Supplement: S1 Sample size — (PDF) [file pone.0213344.s001.pdf]

t tests - Means: Wilcoxon signed-rank test (matched pairs)

**Options:**	A.R.E.* method		
**Analysis:**	Compute required sample size		
**Input:**	Tail(s)	=	Two
	Parent distribution	=	Normal
	Effect size dz	=	0,605142
	α err prob	=	0,05
	Power (1-β err prob)	=	0,8
**Output:**	Noncentrality parameter δ	=	2,9567390
	Critical t	=	2,0692921
	Df	=	22,8732415
	Total sample size	=	25
	Actual power	=	0,8080386

With a power of 0,8 and assuming that NB-UVB has an effect on serum calprotectin levels comparable to the difference found between individuals with psoriasis (mean 38 ± 31,7 ng/ml) (n=47) and healthy controls (mean 20 ± 4,4 ng/ml) (n=8) in the pilot study then calculated sample size is 25. (Correlation coefficient set at 0,5). *Asymptotic relative efficiency.
