# Supplementary material for: Observational study on Swedish plaque psoriasis patients receiving narrowband-UVB treatment show decreased S100A8/A9 protein and gene expression levels in lesional psoriasis skin but no effect on S100A8/A9 protein levels in serum
Source: PLoS One. 2019 Mar 13;14(3):e0213344. doi: 10.1371/journal.pone.0213344 (PMC6415841; doi:10.1371/journal.pone.0213344)
Supplement: S1 Table — (PDF) [file pone.0213344.s002.pdf]

Data	Lesional (L)/Non-lesional (NL) skin	Pre NB-UVB/Post NB -UVB	Shapiro-Wilks sig.
S100A8 gene	NL	Pre	0.000
S100A8 gene	L	Pre	0.001
S100A8 gene	NL	Post	0.000
S100A8 gene	L	Post	0,000
S100A9 gene	NL	Pre	0.000
S100A9 gene	L	Pre	0.015
S100A9 gene	NL	Post	0.000
S100A9 gene	L	Post	0.000
S100A8/A9 protein	NL	Pre	0.000
S100A8/A9 protein	L	Pre	0.146
S100A8/A9 protein	NL	Post	0.000
S100A8/A9 protein	L	post	0.000
PASI	-	Pre	0.001
PASI	-	Post	0.000
Target PASI	-	Pre	0.012
Target PASI	-	Post	0.000
