# Supplementary figures and images for: Observational study on Swedish plaque psoriasis patients receiving narrowband-UVB treatment show decreased S100A8/A9 protein and gene expression levels in lesional psoriasis skin but no effect on S100A8/A9 protein levels in serum
Source: PLoS One. 2019 Mar 13;14(3):e0213344. doi: 10.1371/journal.pone.0213344 (PMC6415841; doi:10.1371/journal.pone.0213344)

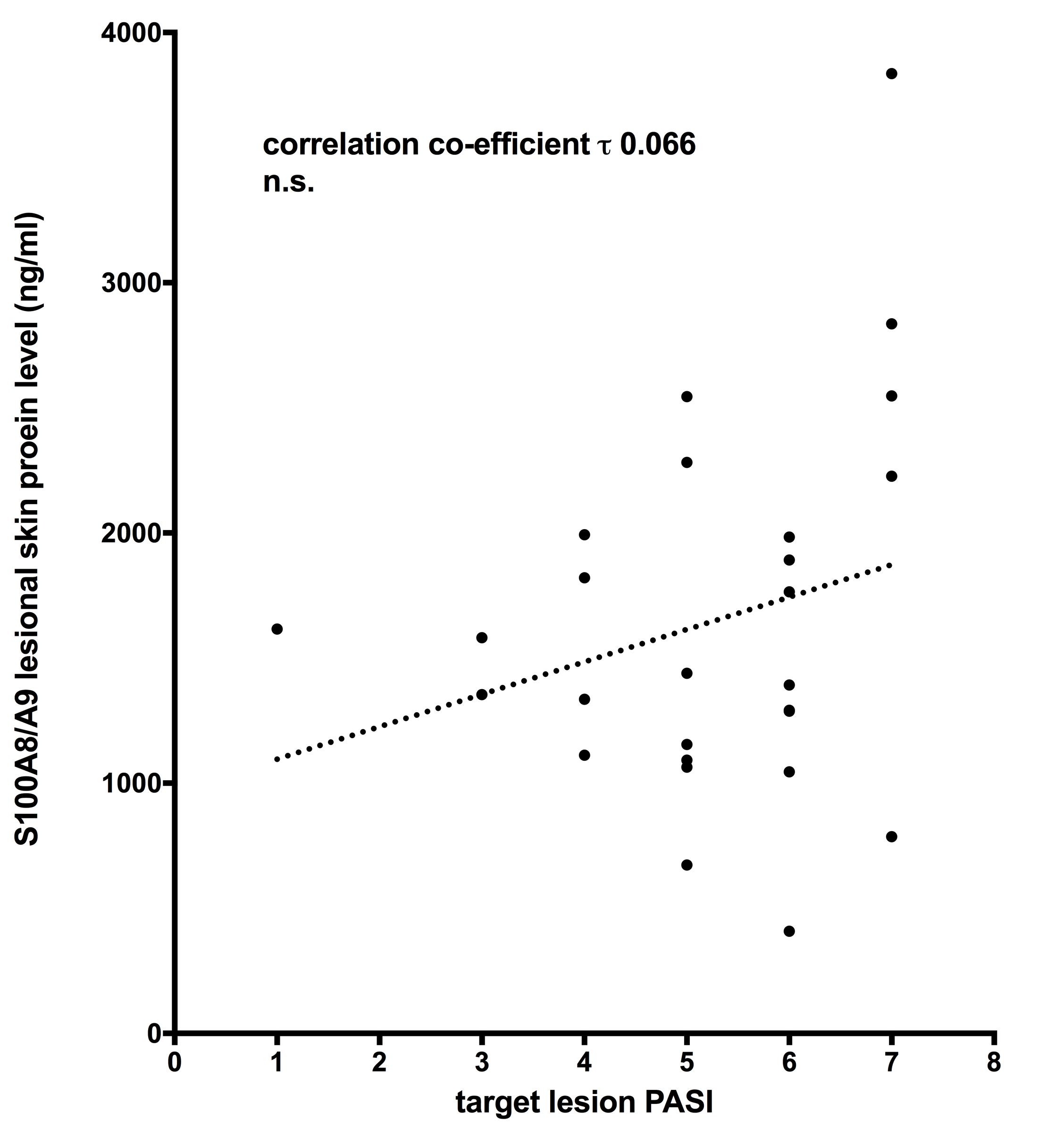

Supplement: S1 Fig — (TIFF) [file pone.0213344.s003.tiff]
