# Supplementary material for: Observational study on Swedish plaque psoriasis patients receiving narrowband-UVB treatment show decreased S100A8/A9 protein and gene expression levels in lesional psoriasis skin but no effect on S100A8/A9 protein levels in serum
Source: PLoS One. 2019 Mar 13;14(3):e0213344. doi: 10.1371/journal.pone.0213344 (PMC6415841; doi:10.1371/journal.pone.0213344)
Supplement: S1 Text — (DOC) [file pone.0213344.s009.doc]

**Method: Selection**

Inclusion criteria are patients diagnosed with chronic plaque psoriasis, age > 18 years attending the division of dermatology at Ryhov hospital who as a result of their disease are planned to upstart UV-treatment.

Exclusion criteria are ongoing systemic psoriasis treatment including UV-treatment, IBD, other skin dermatoses, previous diagnosis of skin cancer and other contraindications for UV treatment, pregnancy, systemic inflammatory disease, ongoing anti-inflammatory medication, exposure to intense UV exposure 2 weeks prior to study start (i.e. vacation, tanning salon), chronic infectious disease.

Subjects will receive written study information prior to recruitment and will have to sign a document proving informed consent. In return subjects will gain an economic compensation of 500 SEK at the end of the study.

**Method: Intervention and tissue sampling**

Patients are treated with narrowband UVB (311nm, TL01) at our outpatient clinic for a total of 25 sessions or until resolution of the underlying disease (3 times per week) according to the clinics standard treatment guidelines. 3,5 ml blood is collected before the first, 5th, 10th, 15th, 20th and 25th session using BD Vacutainer® SST™ Serum Separation Tubes (BD Diagnostics). At the start of treatment a reference plaque is selected. PASI is monitored for this plaque as well as for the skin as a whole at start and before the 5th, 10th, 15th, 20th and 25th session.

The reference plaque should not be exposed to topical treatment 2 weeks before or during the study to avoid confounding-factors. Topical treatment is restricted to moisturizing cream and mometason 0,1%. The latter applied to a maximum of 30% of the total BSA. Mometason is chosen because of minimal systemic uptake(13).

Two 2mm punch biopsies of lesional skin (reference plaque) and two 2mm punch biopsies from healthy skin from corresponding anatomical sites are performed before upstart of TL01 treatment and before the last TL01 session. These biopsies are performed to enable protein and mRNA expression analysis of healthy and lesional skin. Biopsies for PCR analysis are placed in RNAlater and stored at 4 ̊C for 24 hours, surplus RNAlater is then removed before transferring specimens to -196 ̊C before analysis. Biopsies for protein analysis are stored at – 70 ̊C until analysis is performed.

**Method: Data collection**

**S100A8/A9 heterocomplex protein analysis**

Blood samples are inverted five times, allowed 30 minutes clotting time, and centrifuged for 10 minutes at 1000-1300 RCF (g) in a swing bucket centrifuge. 200 µl serum is removed from each blood sample and analysed to obtain the level of S100A8/A9 in blood using Phadia 250 and EliA Calprotectin assay (Thermo Fischer Scientific).

Lesional and healthy skin biopsies are homogenised in lysis buffer (containing 600 µl RIPA buffer, 6 µl PMFS in DMSO, 6 µl protease inhibitor cocktail and 6µl sodium orthovanadate) using a 7mm stainless steel bead and TissueLyser II (Qiagen, Hilden, Germany) set at 30Hz for 3+3 minutes . The lysate is placed on ice for 30 min and then centrifuged at 14,000g for 8 min.

Total protein content of the supernatant fluid is determined using the Bradford protein assay (Bio-Rad Laboratories, CA, USA). 5µl of supernatant fluid is diluted 1/10 by adding 45µl PBS pH 7,4. A series of protein standards are prepared using Quick Start Bovine Serum Albumin Standars – 500 – 0206 2mg/ml (Bio-Rad Laboratories) adding PBS pH 7,4. Bio Rad Dye Reagent is added to standards, blank and supernatant samples and spectrophotometry is performed using Tecan Sunrise (Tecan) at 620 nm using Bradford Quickstart Magellan software.

From lesional skin 20µl supernatant fluid is added to 180µl EliA Sample Diluent (art. nr. 83-1023-01Thermo Fischer Scientific) before S100A8/A9 is analysed using Phadia 250 and EliA Calprotectin assay (Thermo Fischer Scientific). From healthy skin 200µl supernatant fluid is analysed for S100A8/A9 using Phadia 250 and EliA Calprotectin assay (Thermo Fischer Scientific).

**S100A8/A9 expression analysis, PCR**

Lesional and healthy skin biopsies are are homogenized in 350 µl RLT buffer using Tissue Ruptor Disposable Probes (art. No. 990890 Qiagen). RNA is extracted using Qiagen RNeasy Mini Kit (no. 74104). For each sample RNA concentration is determined using Nanodrop Spectophotometer ND-1000 (Nanodrop technologies) and RNA integrity is assessed using Agilent 2100 Bioanalyzer and Agilent RNA 6000 Nano Kit (no. 5067-1511) (Agilent Technologies).

cDNA is synthesized using High Capacity cDNA reverse transcription kit with RNase inhibitor (no. 4374966) (Life technologies – Invitrogen).

**Metod: Data analysis**

To analyse differences between the groups the ANOVA test and Kruskal-Wallis non parametric test will be used. Further we will perform Friedman´s ANOVA to identify changes within subjects and between groups based on treatment results (75%PASI change). Correlation between PASI and S100A8/A9 levels in skin and serum will be assessed using Spearman´s ranking.
