# Supplementary material for: Observational study on Swedish plaque psoriasis patients receiving narrowband-UVB treatment show decreased S100A8/A9 protein and gene expression levels in lesional psoriasis skin but no effect on S100A8/A9 protein levels in serum
Source: PLoS One. 2019 Mar 13;14(3):e0213344. doi: 10.1371/journal.pone.0213344 (PMC6415841; doi:10.1371/journal.pone.0213344)
Supplement: S2 Text — (DOCX) [file pone.0213344.s010.docx]

S10 Differences between study protocol for ethical committee and final protocol

- In the (S1 Text - original protocol) samples were to be obtained before 5^th^, 10^th^, 15^th^ , 20^th^ and 25^th^ NB-UVB session. This would mean that first samples would have been taken after 4 completed treatments and the next after another 5 completed treatments. This was due to a mental lapsus in the planning. Of course sampling should preferably be equidistant from each other when it comes to intervention (NB-UVB changes).
- The method of homogenizing skin biopsies for protein analysis was in the final protocol automatized (using bead beater) so that all biopsies would be treated equal minimizing a possible source of error.
